# Evaluation of a Possible Association Between Severity of Allergic Rhinitis and the Level of Depression in Patients in a Tertiary Care Hospital in South India: A Cross-Sectional Study

**DOI:** 10.7759/cureus.39809

**Published:** 2023-05-31

**Authors:** Joemol John, Nishanth Savery, Prabu Velayutham, Mathan K, Prem Davis

**Affiliations:** 1 Otolaryngology - Head and Neck Surgery, Sri Venkateshwaraa Medical College Hospital and Research Centre, Puducherry, IND; 2 Otolaryngology - Head and Neck Surgery, Aarupadai Veedu Medical College and Hospital, Puducherry, IND; 3 Psychiatry, Sri Venkateshwaraa Medical College Hospital and Research Centre, Puducherry, IND

**Keywords:** hamilton depression scale, aria, severity, depression, allergic rhinitis

## Abstract

Introduction

Allergic rhinitis is one of the most common diseases in the world. It affects all people irrespective of age, sex and race. Allergic rhinitis leads to the development of social and interpersonal problems and loss of productivity which in turn causes depression. The depression was an underestimated iceberg phenomenon in allergic rhinitis patients.

Objective

The study evaluates the association between the severity of allergic rhinitis and the level of depression in patients attending tertiary care hospitals in south India.

Methodology

This cross-sectional study was conducted among 250 patients with allergic rhinitis. All the patients were subjected to the semi-structured questionnaire. And the severity of allergic rhinitis has been made based on the allergic rhinitis, and its impact on asthma classification and depression has been diagnosed and classified based on the Hamilton depression rating scale. And the association between allergic rhinitis and depression has been evaluated with the chi-square test.

Results

Two hundred fifty patients participated in the study, with a mean age of 33+/-2. Surprisingly the prevalence of depression among the allergic rhinitis patient was 88%. Most of them suffered from mild depression based on the Hamilton depression rating scale. A significant association was seen among allergic patients with age, gender, smoking status, locality, socioeconomic status, and co-morbidities. And the study shows the severity of allergic rhinitis is directly related to the severity of depression with a significant association.

Conclusion

Depression is one of the underestimated and under-treated problems in today's world. This study concludes that the severity of allergic rhinitis directly and significantly correlates with the severity of depression. The prevalence and intensity of depression should be evaluated and appropriately treated in patients with allergic rhinitis to improve the quality of life.

## Introduction

Allergic rhinitis (AR) is one of the most common diseases in the world and has a disease prevalence of 0.8 to 39.7% in various parts of the world. About 20 to 30% of the people in India suffer from allergic diseases and the true prevalence of allergic rhinitis is not known [1]. Allergic rhinitis is characterised by inflammatory changes in the nasal mucosa caused by exposure to inhaled allergens [2]. It affects everyone irrespective of age, sex and race [2]. Allergic Rhinitis and its Impact on Asthma (ARIA) published the guidelines for AR and revised them in 2008, and allergic rhinitis is divided based on the symptom duration and severity. Based on this newer classification, AR has been classified into mild intermittent AR, mild persistent AR, moderate-severe intermittent AR and moderate-severe persistent AR [2].

The symptoms of allergic rhinitis are sneezing and nasal itching, rhinorrhoea and nasal block. It is also associated with co-morbidities like conjunctivitis, chronic otitis media, sleep apnoea, hyposmia and bronchial asthma. Patients also had uncommon symptoms like throat itching, watering of eyes, headache, and aural block. These will directly impact the quality of life and the mental health of the patients [3]. Due to the frequency of symptoms, the patient often feels embarrassed to be in groups and can be treated as a social outcast. Patients can become fatigued frequently and have a diminished ability to concentrate or think [4].

The pathogenesis of the development of depression in AR remains unclear. Apart from this direct psychological association leading to depression, there are many studies that showed that the cytokines which are produced during the pathogenesis of AR also lead to the development of depression. Certain animal models had demonstrated that the passage of the pepties and interleukins which are developed during the pathogenesis of the AR travel to the brain from the nose [5]. An interesting result from a meta-analysis of 24 studies showed that there was a higher concentration of tumor necrosis factor (TNF) and interleukin-6 (IL-6) seen in the depressed patients than the normal patients [5]. Studies also showed a strong association between inflammatory markers and depression [6]. 

Sleep disturbances, which were noted in AR patients, also lead to the development of depression. It was noted that 8% of seasonal AR and 68% of perennial AR patients had sleep disturbance [7]. AR patients had sleep disturbance through obstructive symptoms and due to the production of cytokines which in turn leads to increase of nasal secretions. This sleep disturbance is the major factor among AR patients in the development of various psychiatric disorders like anxiety disorder and bipolar mood disorder and for the development of suicidal thoughts [7]. 

Antihistamines used in treating allergic rhinitis can be beneficial in patients with insomnia or detrimental, causing worsening sedation, decreased productivity and increased risk of accidents. Patients' compliance to long-term and proper use of nasal sprays is poor, leading to periodic exacerbations of symptoms and chronicity of the disease [8].

Major depression is a worldwide public health problem that affects approximately 5% of the global population. Worldwide the prevalence of depression among allergic rhinitis was estimated to be between 20 to 40% [4]. But most depression among allergic rhinitis patients in India is unknown due to underreporting of cases. This psychiatric disorder represents a significant cause of impairment, disability, economic burden, and mortality [9]. Allergic rhinitis profoundly impacts patient's quality of life and productivity and has been shown to have a significant economic burden. The aim of this study has to demonstrate the prevalence of depression among AR patients. 

## Materials and methods

Study design

This cross-sectional study was conducted among allergic rhinitis patients in a tertiary care centre (Sri Venkateshwaraa Medical College Hospital and Research Centre) in Puducherry. All the patients were provided with a semi-structured questionnaire.

Sampling

Considering the proportion of allergic rhinitis among the general population is 34.5, taking 5% as alpha error and 6% as absolute margin error, the calculated sample size was 241. All the patients were selected by convenient sampling technique.

Inclusion criteria

Patients diagnosed with allergic rhinitis with age between 18 and 60 years of age were included in the study.

Exclusion criteria

Patients diagnosed with other forms of rhinitis except for allergic aetiology who are diagnosed with any psychological disorder or on any psychiatric medications and with other co-morbidities based on history and previous treatment records were excluded from the study.

Data collection

The questionnaire's ambiguity and comprehensibility was tested among 5% of the patients before the initiation of the original study and the questionnaire was changed accordingly. Pre-tested students were excluded, and institutional experts validated the questionnaire. After obtaining approval from Institute Ethics Committee and informed written consent, all the participants were asked to answer all the questions in the self-administered questionnaire, maintaining anonymity and the confidentiality of the data was maintained.

Study variables 

The semi-structured questionnaire consisted of three parts. The first part consisted of the basic demographic details like age, gender, marital status, course, year, institution type, place of matriculation and socioeconomic status based on a modified BG Prasad scale [10], religion and residence. The second part consisted of symptoms of allergic rhinitis with troublesome symptoms. The diagnosis and classification of allergic rhinitis have been made in this section based on the ARIA classification [2]. The third part consisted of the Hamilton depression rating scale [11], the most widely used clinician-administered depression assessment scale in the world. It comprised 17 items. For all things, a score of zero (0) indicates the absence of that particular symptom. Classification of symptoms which may be difficult to obtain can be scored as 0 - absent: 1 - doubtful or trivial: 2 - present. Classification of symptoms were graded as 0 - absent; 1 - mild; 2 - moderate; 3 - severe; 4 - incapacitating. The higher the total score, the more severe the depression. The level of depression was made based on the cumulative score of the individual items. A score of 0 to 7 is taken as normal and score of 8 to 13 as mild depression, the score of 14 to 18 and 19 to 22 as moderate depression and severe depression respectively. And a score of more than 23 was taken as very severe depression.

Data analysis

All the data were entered via Epi-Data software and were analysed using the Statistical Package for Social Sciences (SPSS) version 20 software (IBM Corp., Armonk, NY, USA). Chi-square and correlation analysis was used to find the association between the various variables of the study.

Ethical approval

The study protocol was approved by the Internal Human Ethics Committee of the Sri Venkateshwaraa Medical College Hospital and Research Centre, Ariyur, Puducherry, with reference number 122/SVMCH/IEC-Cert/Nov21.

## Results

A total of 250 patients with allergic rhinitis participated in the study. The mean age of the study participants was 33+/-2. Regarding the basic demographic details, nearly 44% of the patients belonged to 30 to 40 years of age, 22% belonged to 40 to 50 years of age, and 19% and 15% of the participants were in their 20 to 30s and 50 to 60 years age group respectively as in Table 1. Nearly 51% of the study participants were female, and 49% were males.

**Table 1:** Socio-demographic characteristics of the study population (n=250)

Age (in years)	Frequency	Percentage
20-30	47	19
30-40	110	44
40-50	56	22
50-60	37	15
Gender		
Male	123	49
Female	127	51
Smoking status		
Non- Smoker	173	69
Smoker	77	31
Locality		
Rural	163	65
Urban	87	35
Socio-Economic Status		
More than Rs. 7533 per month (Class I)	18	7.3
Rs 3766-Rs. 7533 per month (class II)	24	9.4
Rs. 2260 – Rs. 3765 per month (class III)	151	60.5
Rs 1130 – Rs. 2259 per month (class IV)	57	22.7
Co-morbidities		
Asthma	88	35.2
Coronary Artery Disease	21	8.4
Hypertension	43	17.2
Diabetes	59	23.6
Thyroid diseases	23	9.2

Regarding the smoking status of the patients with allergic rhinitis, nearly 69% of the study participants were non-smokers, and 31% were smokers. Sixty-five percent of the study participants reside in rural areas, and only 35% are from rural areas. More than half of the patients, 60.5%, belonged to class III socioeconomic status according to the modified BG Prasad classification, 22.7% in class IV socioeconomic status, and only 9.4% and 7.3% in class IV socioeconomic status, class II and class I socioeconomic status respectively. Regarding the patient's co-morbidities, about 35.2% had asthma, 23.6% had diabetes, 17.2% of the study participants suffered from hypertension, 9.2% had thyroid diseases, and only 8.4% of the study participants had coronary artery disease, as in Table 1.

**Figure 1:**
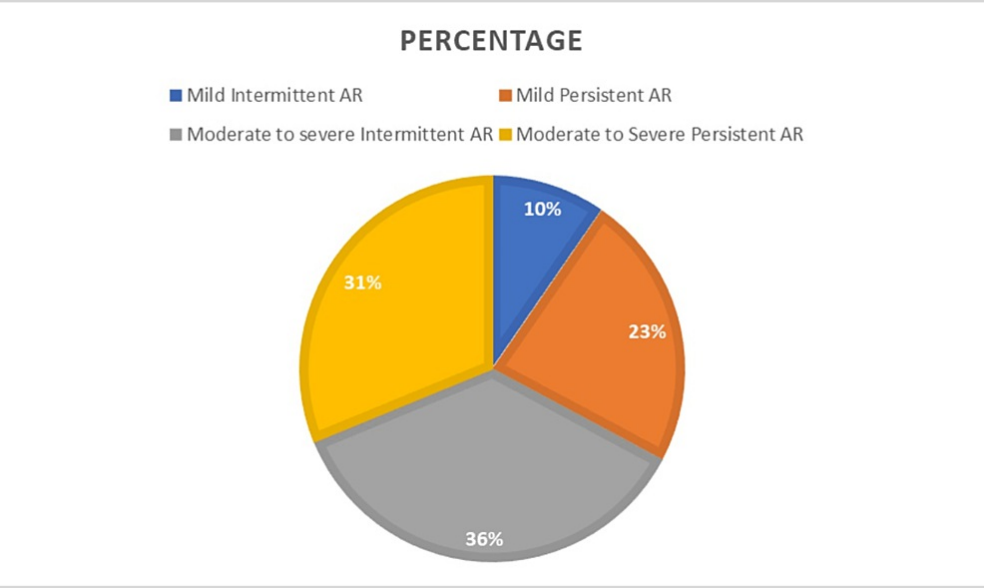
Percentage distribution of patients with severity of allergic rhinitis based on ARIA classification (n=250) AR: allergic rhinitis, ARIA: Allergic Rhinitis and its Impact on Asthma

Based on the ARIA classification, nearly 36% of the people belonged to moderate to severe intermittent allergic rhinitis, 31.2% belonged to moderate to severe persistent allergic rhinitis, and 23.2% were in the mild persistent allergic rhinitis category. Only 9.6% were in the mild intermittent allergic rhinitis category, as in Figure 1.

**Figure 2:**
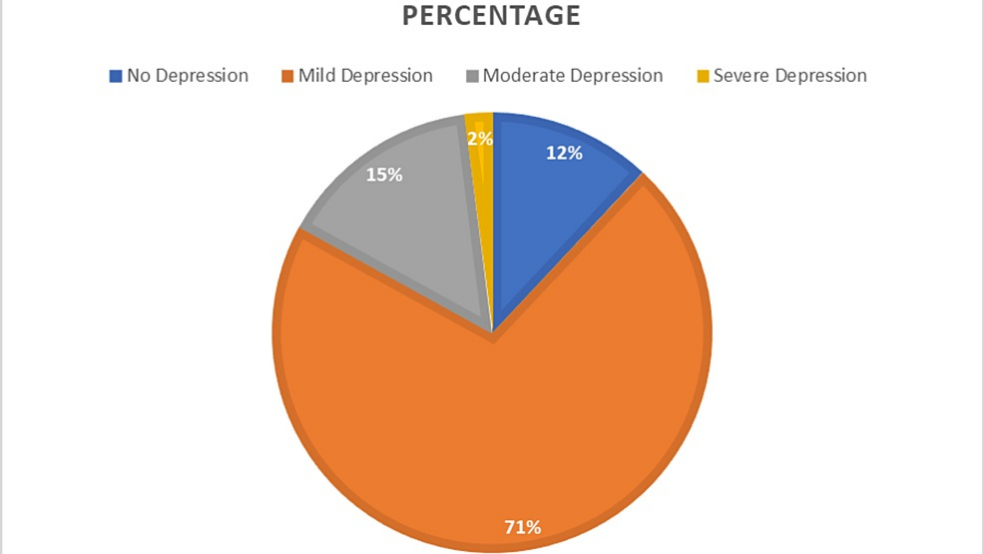
Percentage distribution of allergic rhinitis patients with severity of depression (n=250)

Surprisingly, 88% of the people with allergic rhinitis suffered from some form of depression, according to the Hamilton depression rating scale. Nearly three-fourths (71%) were in the mild category, followed by 15% in moderate intensity. Fortunately, only 2% of the study participants had severe depression, as in Figure 2.

On comparing the various demographic details of the allergic rhinitis patients with depression, 95.5% of the allergic rhinitis patients in the 30-to-40-year age group had depression, followed by 87.5% in the 40-to-50-year age group. Nearly 91.9% and 68% of allergic rhinitis patients had depression in their 50 to 60s and 20 to 30s, respectively. Almost 96%, of the female allergic rhinitis patients and 81.3% of male allergic rhinitis patients had depression. Surprisingly,93.6% of the non-smoker had depression, and only 75.3% of allergic rhinitis patients who smoke had depression. 94.4% of the allergic rhinitis patients coming from rural areas had depression compared to 75.8% who had depression in urban areas. On evaluating the socioeconomic status, 99.3% of patients with class III socioeconomic status had depression. Compared to 98.2% in class IV, 54.1% in type II and only 5.6% in category I had depression. It is exciting to see that 88.6% of the allergic rhinitis patient with asthma had depression, 20% of the diabetes patients, 76.7% of the hypertension patient and 95.2% of the coronary artery disease patient had as in Table 2.

**Table 2:** Association of characteristics of allergic rhinitis patients with the presence of depression (n=250)

Characteristics	Frequency	Depression	
		Present	Absent
Age (in years)			
20-30	47	32 (68)	15 (32)
30-40	110	105 (95.5)	5 (4.5)
40-50	56	49 (87.5)	7 (12.5)
50-60	37	34 (91.9)	3 (8.1)
Gender			
Male	123	100 (81.3)	23(18.7)
Female	127	122 (96)	5 (4)
Smoking status			
Non-Smoker	173	162 (93.6)	11 (6.4)
Smoker	77	58 (75.3)	19 (24.7)
Locality			
Rural	163	154 (94.4)	9 (5.6)
Urban	87	66 (75.8)	21 (24.2)
Socio-Economic Status			
More than Rs. 7533 per month (Class I)	18	1 (5.6)	17 (94.4)
Rs 3766-Rs. 7533 per month (class II)	24	13 (54.1)	11 (45.9)
Rs. 2260 – Rs. 3765 per month (class III)	151	150 (99.3)	1 (0.7)
Rs 1130 – Rs. 2259 per month (class IV)	57	56 (98.2)	1 (1.8)
Co-morbidities			
Asthma	88	78 (88.6)	10 (11.4)
Coronary Artery Disease	21	20 (95.2)	1 (4.8)
Hypertension	43	33 (76.7)	10 (23.3)
Diabetes	59	50 (84.7)	9 (15.3)
Thyroid diseases	23	12 (52.1)	11 (47.9)

And on comparing the association of severity of allergic rhinitis with the presence of depression, nearly 94.4% of the patients in moderate to severe intermittent allergic rhinitis patients had depression, all the allergic rhinitis patients (100%) in moderate to severe persistent allergic rhinitis had depression, 87.9% in the mild persistent allergic rhinitis category and only 25% in mild intermittent allergic rhinitis category had depression with a significant p-value of <0.01 as in Table 3. And on comparing the severity of allergic rhinitis with the severity of depression, in most mild intermittent allergic patients, only 16.7% had mild depression. In the mild persistent allergic rhinitis, 60.3% had mild depression, followed by 29.3% had moderate depression. In the moderate to severe intermittent allergic rhinitis patients, nearly 86.7% had mild depression, 6.7% had moderate depression, and only 2.2% had severe depression. Of the moderate to severe persistent allergic rhinitis patients, 76.9% had mild depression and 19.2% of patients had moderate depression, and only 2.6% had severe depression, as in Table 4.

**Table 3:** Association of the severity of allergic rhinitis with depression (n=250)

Allergic Rhinitis	Frequency	Depression		Chi-square tests	p-value
		Present	Absent		
Mild Intermittent AR	24	6 (25)	18 (75)	104.38	<0.01
Mild persistent AR	58	51 (87.9)	7 (12.1)		
Moderate to severe Intermittent AR	90	85 (94.4)	5 (5.6)		
Moderate to severe persistent AR	78	78 (100)	0 (0)		

**Table 4:** Association of the severity of allergic rhinitis (AR) with the severity of depression (n=250)

Category of AR	Total No of AR patients	Severity of Depression			
		No Depression	Mild Depression	Moderate Depression	Severe Depression
Mild Intermittent AR	24	20 (83.3)	4 (16.7)	-	-
Mild persistent AR	58	5 (8.6)	35 (60.3)	17 (29.3)	1 (1.72
Moderate to severe Intermittent AR	90	4 (4.4)	78 (86.7)	6 (6.7)	2 (2.2)
Moderate to severe persistent AR	78	1 (1.3)	60 (76.9)	15 (19.2)	2 (2.6)

## Discussion

This study evaluates the association of the severity of allergic rhinitis with the severity of depression. In this study, the severity of allergic rhinitis has been evaluated by the ARIA classification, and the depression severity by the Hamilton depression rating scale. In this study, about 44% of the patients with allergic rhinitis were in the 30-to-40-year age group, followed by 22% in the 40 to 50s age group. This supports the epidemiology of allergic rhinitis, affecting all age groups [2]. And in our study, an equal number of males and females participated, as stated in the Bousquet study. And the study by Karthikeyan et al. [12] also had an equal distribution of the sex as in the study. In our study, about 31% of the patients with allergic rhinitis are smokers. It is due to the fact that the allergens in the smoke induce immunological reactions, leading to the development of allergic rhinitis. The results are consistent with the study by Gomez et al., where 25% of smokers have allergic rhinitis [13]. In our study, allergic rhinitis affects all socioeconomic status people and is more commonly seen among the lower classes like class III and IV, than the higher classes. The reason is that people in higher classes are more readily approachable to the medical facilities for treating diseases than the lower socioeconomic people. The study by Hardly et al. also shows it affects all socioeconomic status people [14]. The prevalence of allergic rhinitis is more commonly seen in asthmatic patients, 35.2%. Similar results were also noted in the study by Bousquet et al. [15]. In our research, most patients were in the moderate to severe allergic rhinitis category. This may be because the mild category of patients has intermittent symptoms that are manageable. They would not have reported to the health care facility very often as the moderate to severe category patients.

In this study, depression is more commonly seen among female allergic rhinitis patients than among males, which is consistent with the results from the study by Kim et al. [16]. And astonishingly, in this study, non-smokers (93.6%) are more commonly affected by depression than smokers. The study by Fluharty et al. studied the effect of cigarette smoking on depression. It concluded the study with an exciting statement that smoking leads to depression and anxiety, and likewise, depression and anxiety lead to smoking, and they had a bidirectional relationship [17]. In our study, 99.3% of class III socioeconomic status patients had depression, followed by class IV (98.2%). People in the upper socioeconomic classes had very low depression rates. Similar results were also noted in the study by Ahmed et al., where the lower socioeconomic status people are more prone to depression than higher class people [18]. This may be because the lower socioeconomic people didn't get proper treatment for the treatment of allergic rhinitis, and allergic rhinitis has a significant impact on the quality of life of the patient, so they are more prone to develop depression than those of the higher socioeconomic status people [19]. Our study also shows that patients with co-morbidities are more prone to developing depression. Similar results were also noted in the study by Ahmed et al., where people with non-communicable diseases were more prone to develop depression than those without co-morbidities [18].

In our study, the patients with moderate to severe intermittent allergic rhinitis and moderate to severe persistent allergic rhinitis had more depression rates than those with mild intermittent and mild persistent allergic rhinitis. Similar results were also noted in the study of Roxbury et al., where allergic rhinitis is more commonly seen among patients with a severe form of depression than the milder one.

Limitations of the study

As the sample size is very small, the conclusion cannot be generalized. And depression can be caused by multiple other confounding factors like religion, matriculation status, medical co-morbidities, socio-demographic profile etc. But due to resource restriction, the following parameters were not added to our study. Though the questionnaire is a screening questionnaire, it has been used in multiple studies as a diagnostic and prognostic tool but still the questionnaire is not validated as a diagnostic tool.

## Conclusions

This study evaluates the allergic rhinitis severity with the severity of depression. The results suggest that screening of allergic rhinitis with other co-morbidites is associated with risk of depression. On further evaluation, the severity of allergic rhinitis is also directly proportional to the severity of the depression among the patients. However, future studies are required to increase our understanding of the association between allergic rhinitis and the development of depression to prevent it from affecting people and to increase the patient's quality of life.
